# P04.53. Factors related to use of both western medicine and complementary and alternative medicine among patients with chronic diseases in South Korea

**DOI:** 10.1186/1472-6882-12-S1-P323

**Published:** 2012-06-12

**Authors:** B Choi, B Lim, D Han

**Affiliations:** 1 Pusan National University School of Korean Medicine, Yangsan, Gyeongnam, Republic of Korea; 2 Hanyang University College of Medicine, Seoul, Republic of Korea

## Purpose

This study aims to examine the behavior of patients with chronic diseases using both western medicine (WM) and complementary and alternative medicine (CAM), and the factors affecting CAM utilization.

## Methods

A cross-sectional study was carried out in three long-term care hospitals in Korea using a random sample of 620 adult patients with chronic diseases. The measures were CAM use and recognition of CAM. The factors affecting the use of CAM were analyzed statistically.

## Results

Of 423 respondents, 79.0% used CAM adjunctively during the hospitalization period. The most frequent type of CAM modality used was a package of herbal medicine and acupuncture (91.3%). Of the patients using CAM, 34.4% had musculoskeletal disorders and 16.1% had hypertension. Those aged 40-59 used CAM more than those aged 20-39 (OR = 4.58, 95% CI: 1.92-10.92). Males (OR = 0.33, 95% CI: 0.18-0.60) and people who had a spouse (OR = 0.26, 95% CI: 0.13-0.52) used CAM less.

## Conclusion

In Korea, most hospitalized patients with chronic diseases used CAM modalities adjunctively. Age, disease and sex were important factors affecting CAM use. The guidelines for proper CAM use should be provided to hospitalized patients.

